# Facile Synthesis of Holmium-Based Nanoparticles as a CT and MRI Dual-Modal Imaging for Cancer Diagnosis

**DOI:** 10.3389/fonc.2021.741383

**Published:** 2021-08-26

**Authors:** Tianqi Zhang, Mo Deng, Lei Zhang, Zerun Liu, Yang Liu, Shuyan Song, Tingting Gong, Qinghai Yuan

**Affiliations:** ^1^Department of Radiology, The Second Hospital of Jilin University, Changchun, China; ^2^Department of Clinical Laboratory, The Second Hospital of Jilin University, Changchun, China; ^3^Department of Neurology, The Second Hospital of Jilin University, Changchun, China; ^4^Department of Clinical Pharmacy, Jilin University School of Pharmaceutical Science, Changchun, China; ^5^State Key Laboratory of Rare Earth Resource Utilization, Changchun Institute of Applied Chemistry, Chinese Academy of Sciences, Changchun, China

**Keywords:** nanomaterial, cancer, diagnosis, dual-model imaging, contrast agent

## Abstract

The rapid development of medical imaging has boosted the abilities of modern medicine. As single modality imaging limits complex cancer diagnostics, dual-modal imaging has come into the spotlight in clinical settings. The rare earth element Holmium (Ho) has intrinsic paramagnetism and great X-ray attenuation due to its high atomic number. These features endow Ho with good potential to be a nanoprobe in combined x-ray computed tomography (CT) and T_2_-weighted magnetic resonance imaging (MRI). Herein, we present a facile strategy for preparing HoF_3_ nanoparticles (HoF_3_ NPs) with modification by PEG 4000. The functional PEG-HoF_3_ NPs have good water solubility, low cytotoxicity, and biocompatibility as a dual-modal contrast agent. Currently, there is limited systematic and intensive investigation of Ho-based nanomaterials for dual-modal imaging. Our PEG-HoF_3_ NPs provide a new direction to realize *in vitro* and *vivo* CT/MRI imaging, as well as validation of Ho-based nanomaterials will verify their potential for biomedical applications.

## Introduction

Imaging technology plays an important role in modern medicine due to its ability to provide noninvasive but detailed information of anatomical structure and functional activities during the progress of a disease. However, single-model imaging methods cannot always meet the criteria for diagnosing co2mplex diseases (1, 2); thus, multi-modal imaging has become the new direction for imaging technology development (3–6). There are two main means to achieve multi-modal imaging (1): endow one device with multiple imaging capabilities; or (2) construct multi-modal contrast agents (CAs) for diagnosis. Conflicts between various methods and high costs have limited the feasibility of the first method. Hence, researchers are focusing their efforts on developing multi-modal CAs that can be widely used in magnetic resonance imaging (MRI), X-ray computed tomography (CT), as well as fluorescence imaging (FI) (7–13). Due to the penetrative limitation of FI *in vivo*, CT and MRI are more commonly applied for clinical diagnosis (14–17). CT is the most cost-effective examination and offers strong X-ray penetration, which has very high resolution for bone and calcification but poor resolution for soft tissue. Notably, MRI can remedy this shortcoming. Therefore, the combination of CT and MRI can significantly improve diagnostic ability (2, 18–24).

**Scheme 1:**
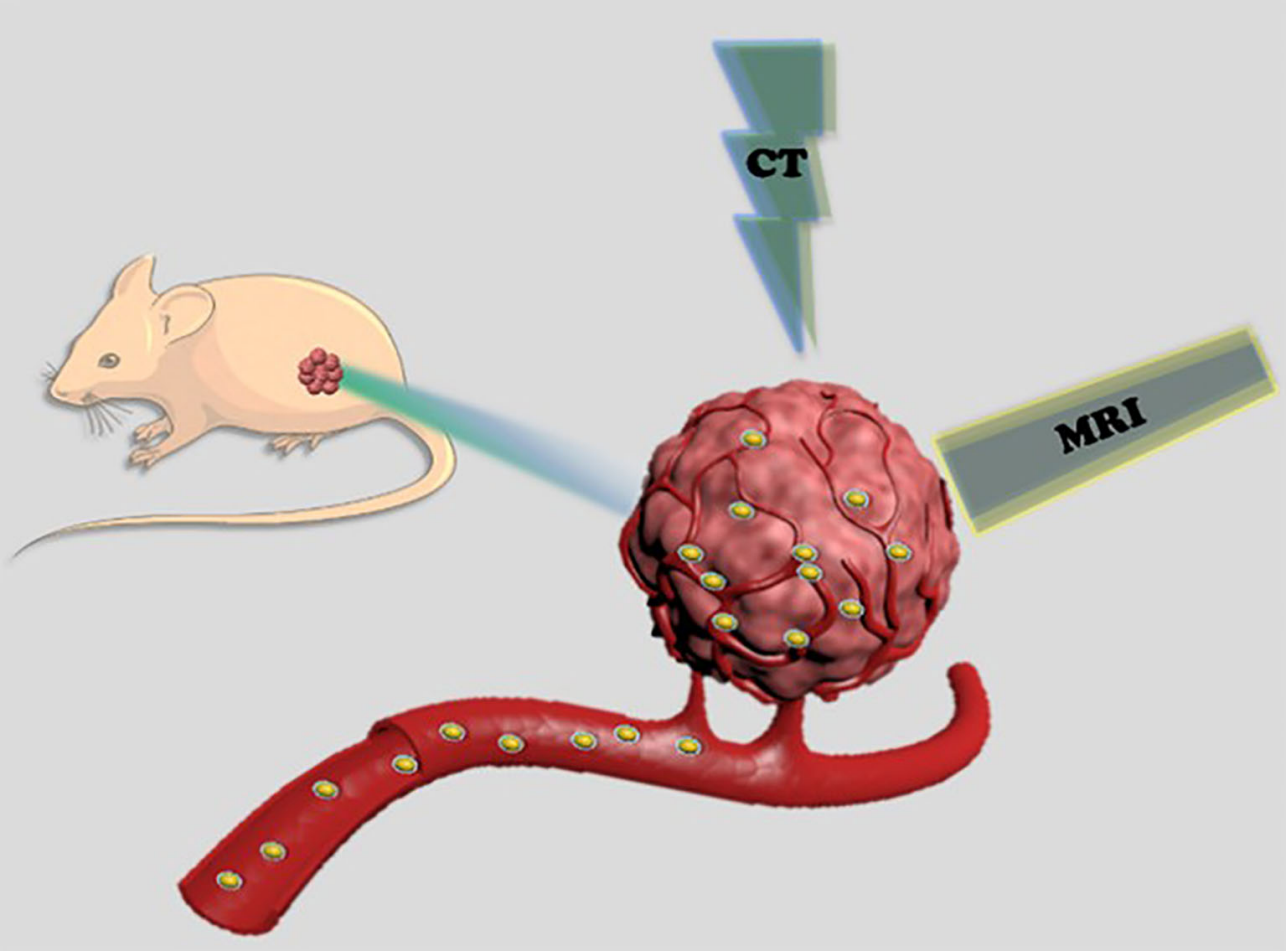
Schematic illustration of PEG-HoF_3_ NPs for enhanced CT and T_2_-weighted duel-model imaging.

At present, CT and MRI examinations are used independently in clinical settings. Small iodinated molecule nanomaterials are routinely applied as CT CAs to assist in disease diagnosis. Due to the lack of X-ray absorption, a very large amount of such CAs for intravenous injection is needed to meet the contrast requirement, usually 80-150 ml per adult for angiography (25, 26). Such a high dose is not only likely to cause allergy, but it can also lead to irreversible renal damage (27–29). Furthermore, as small molecular iodide is quickly excreted by the kidney, its short circulation lifetime can affect imaging quality for certain diseases. Similarly, MRI has its own limitations. Gd-chelates are the most commonly used commercial MRI CAs in clinical settings. However, Gd-based CAs can accumulate in the central nervous system and lead to renal fibrosis (30). Another notable disadvantage of MRI is that the optimum magnetic field strength for current Gd-based CAs is less than 1.0 T (31), whereas the trend in clinical MRI is to use ultra-high magnetic fields of 7.0 T or even higher because of the better imaging quality (32). 7.0 T MRI has been available in the market since 2017, and ultra-high field strength MRI equipment will become mainstream in the future. As a result, several researchers have been focusing on developing new CAs to suit this powerful visualization tool. Tb, Ho, and Dy are the elements with the largest magnetic moments in the periodic table and can cause considerable transverse relaxation of hydrogen protons in free water. Thus, Tb, Ho, and Dy-based materials are the best choices for ultra-high field T_2_ CAs (33–36). Against the background of continuous pursuit of ultra-high field intensity MRI, research on MRI CAs based on large magnetic moment elements is likely to experience rapid growth in the near future. Thus, we developed a facile strategy to construct control-sized PEGylated HoF_3_ nanoparticles (PEG-HoF_3_ NPs) as a dual-modal imaging CA. **Scheme 1**. illustrates the design of our study. We used a facile one-pot solvothermal approach to obtain Ho-based nanomaterial, which has a high X-ray attenuation and large magnetic moment. PEG-HoF_3_ NPs offer great biocompatibility and low cytotoxicity due to the existence of poly(ethylene glycol) (PEG). As a result, these NPs could be used as CT/MRI dual-modal imaging CAs both *in vitro* and *in vivo*, confirming the potential of Ho-based nanomaterials for bioapplication research.

## Materials and Methods

### Chemicals

HoCl_3_•6H_2_O, poly(ethylene glycol) (PEG, MW = 4000 Da) was obtained from Sigma-Aldrich. NH_4_F and ethylene glycol (EG) were obtained from Beijing Chemicals. Dimethyl sulfoxide (DMSO) were obtained from Sigma-Aldrich. DMEM and FBS were purchased from Gibco.

### Preparation of PEG-HoF_3_ NPs

PEG-HoF_3_ NPs were fabricated *via* a one-pot solvothermal method. Firstly, 0.8 mmol HoCl_3_•6H_2_O was dissolved in 5 mL of EG to form a clear solution. Then, 0.6 g of PEG 4000 was added to 15 mL of EG to form a clear solution. Next, prepared EG with 2.4 mmol NH_4_F added to the mixture of above two solutions. This mixed solutions were vigorously stirred for about 40 min and then transferred to a 50 mL Teflon-lined autoclave and kept at 200°C for 10 h. When the system was cooled down, all collected NPs were washed several times.

### *In Vitro* Cytotoxicity Assessment

Human cervix cancer cells (HeLa) and mouse fibroblast cells (L929) were cultured in DMEM with 10% FBS, 1% penicillin, and 1% streptomycin at a 37°C in a 5% CO_2_ incubator. After incubation in 96-well cell culture plates for 24 h, different concentrations of PEG-HoF_3_ NPs (0, 25, 50, 100, 200, 300 μg/mL) were added to the HeLa cells and L929 cells and incubated for another 24 h. Next, 10 μL (5 mg/mL) MTT was added to 96-well cell culture plates and kept for an additional 4 h at 37°C. Finally, the medium was removed and DMSO was added for 15 min to dissolve the formazan. The absorbance peak at 570 nm was measured by a microplate reader.

### Histological and Hematology Assessment

Kunming mice (18-25 g) were obtained from the Center for Experimental Animals, Jilin University (Changchun, China). All operations are carried out in accordance with relevant national regulations. For histological assessment, 100 μL PEG-HoF_3_ NPs (300 μg/mL) and 100 μL 0.9% NaCl solution were administered to Kunming mice *via* tail vein. After 30 days, Major organs and tissues (heart, liver, spleen, lung, kidney, and muscle) were collected. All tissues samples were formalin-fixed, paraffin-embedded, and stained with H&E. Tissue sections were observed under an optical microscope (×10). Blood samples were also obtained from the PEG-HoF_3_ NPs and NaCl injected mouse groups for routine blood testing and biochemical indices testing.

### CT Imaging

Different concentration of PEG-HoF_3_ NPs and Iohexol aqueous solutions (I or Ho 0, 0.25, 0.5, 1, 2, 4 mmol/mL) were prepared for *in vitro* CT imaging. When the suitable con-centration was determined, *in vivo* CT images were obtained on Kunming mice. After intraperitoneal anesthesia with chloral hydrate, 100 μL PEG-HoF_3_ NPs (2 mg/mL) were injected into these mice *via* tail vein and images were obtained at different times (0, 1, 2, 6, 12, 24 h). CT was performed using a clinical CT scanner, and the parameters were tube voltage of 120 kVp, tube current of 300 mAs, 0.9 mm thickness, 0.99 pitch, window width of 200 HU, and window level of 45 HU.

### T_2_-Weighted MRI

Different concentrations of PEG-HoF_3_ NPs aqueous solutions (Ho 0, 0.0625, 0.125, 0.25, 0.5, 1 mmol/mL) were prepared for *in vitro* T_2_-weighted MRI. Preliminary preparation was similar to the above steps. MRI was performed on a clinical MR scanner. 100 uL PEG-HoF_3_ NPs (0.8 mg/mL) were injected into mice as above. These mice were scanned pre-injection and 1, 2, 4, 12, and 24 h post-injection. The MR scanner parameters were TR=7279.7 ms, TE=113 ms and FOV=240x240 mm.

## Results and Discussion

### Preparation and Characterization of PEG-HoF_3_ NPs

**Figure 1:**
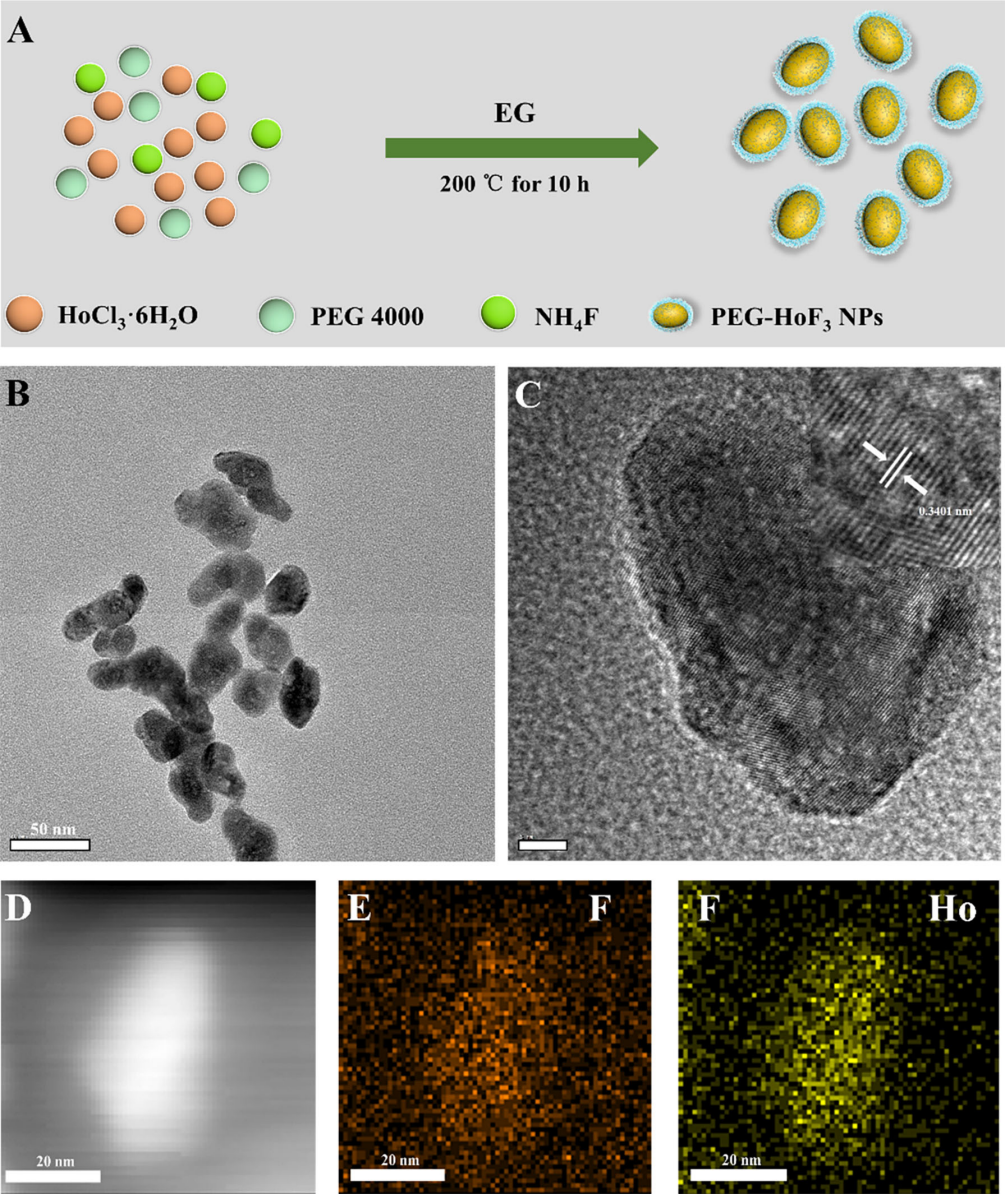
**(A)** The synthetic process of PEG-HoF_3_ NPs; **(B, C)** TEM image of PEG-HoF_3_ NPs, the particles size is about 30-40nm and its lattice fringes;**(D–F)**. HADDF-STEM image of PEG-HoF_3_ and EDS mapping of F, Ho element.

**Figure 2:**
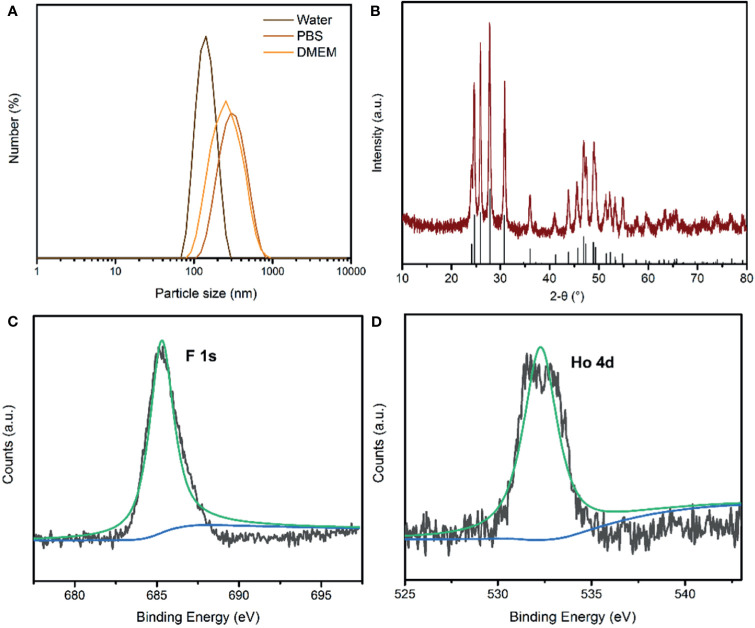
**(A)** The dynamic light scattering (DLS) of PEG-HoF_3_ NPs; **(B)** XRD pattern of PEG-HoF_3_ NPs; **(C, D)** Higher-resolution XPS spectra of Ho 4d and F1s, respectively.

Uniform HoF_3_ NPs were prepared through a one-pot solvothermal method using PEG 4000 as a surfactant, as illustrated in **Figure 1A**. The TEM images demonstrate the good dispersivity of PEG-HoF_3_ NPs, and the existence of Ho and F element is proved by HADDF-STEM image and EDS mapping (**Figures 1B–F**), and the high-resolution TEM image shows that the width of lattice fringes about PEG-HoF_3_ NPs was 0.3401 nm. The SEM image shows the spherical nanoparticles with an average diameter of 38 nm; the diameter of PEG-HoF_3_ NPs was normally distributed in the range of 30–55 nm (**Figure S1**). In order to ascertain the hydrodynamic diameters of PEG-HoF_3_ NPs, the dynamic light scattering (DLS) was performed, and the results show the average hydrodynamic diameter are suitable for using in organism in different solvents (**Figure 2A**). Meanwhile, the Zeta potential of PEG-HoF_3_ NPs was 5.83 mV with the decoration of PEG. These value are suitable for nanomaterial which will be applied *in vivo*. XRD analysis exhibited several strong peaks, which indicated the highly crystalline nanostructure of this material. The diffraction peaks of the nanoparticles can be indexed to orthorhombic HoF_3_, which matches the card (PDF 00-023-0284) in **Figure 2B** (34). XPS spectra verified that the NPs contained Ho 4d, F 1s (**Figures 2C, D**), further confirming the existence of HoF_3_ NPs. The peaks of C and O confirmed the presence of PEG (**Figure S2**). The peak in 3391 cm^-1^ of FTIR spectrum further confirm the existence of PEG (**Figure S3**). Due to the low toxicity and bio-tolerability of PEG, it was approved for use in biopharmaceuticals by the US FDA several years ago (37, 38). Using PEG 4000 to modify the surface of HoF_3_ NPs not only reduced cytotoxicity, but also enhanced dispersibility in water and phosphate-buffered saline (PBS) solution. The picture shows good stability of PEG-HoF_3_ NPs in normal saline and PBS solution (**Figure S4**). There was no obvious Ho^3+^ ion dissociation after long-term dialysis in PBS solution (pH=7.4) with PEG-HoF_3_ NPs.

### Biocompatibility Assessment of PEG-HoF_3_ NPs

**Figure 3:**
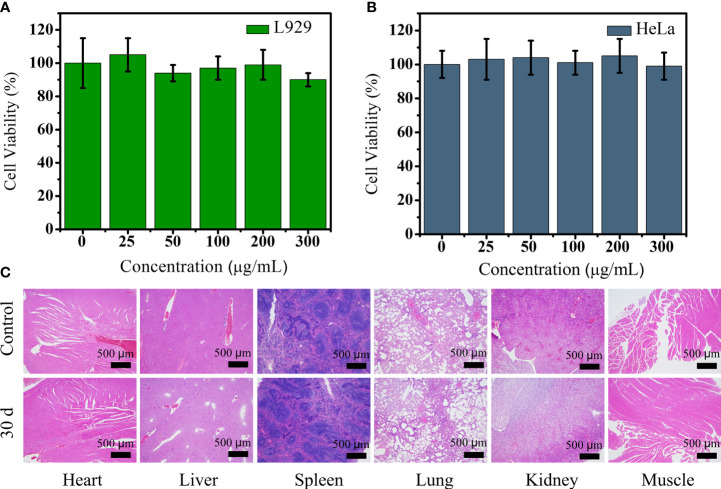
**(A, B)** Viability of L929 and HeLa cells after incubation with different concentrations of PEG-HoF_3_ NPs; **(C)** Tissue sections of 6 major organs from mouse which injected with PEG-HoF_3_ NPs *via* tail vein.

Biocompatibility assessment should be carried out before nanomaterial imaging probes are applied *in vivo*. Due to the lack of data about the application of Ho-based nanomaterial *in vivo*, we carried out a variety of tests to assess the safety of PEG-HoF_3_ NPs. The cytotoxicity of PEG-HoF_3_ NPs was evaluated by the MTT cell proliferation assay. We chose HeLa and L929 cells to evaluate the toxicity of the new Ho-based nanomaterial by observing damage in cancer cells and normal cells, respectively. The cell viability results are depicted in **Figures 3A, B**. The two cell types were exposed to PEG-HoF_3_ NPs at different extracellular concentrations (0-300 μg/mL). As expected, the new PEGylated Ho-based nanoparticles had great biocompatibility. Cell viability was still about 85% of the highest concentration (300 μg/mL) in both cell strains. We further investigated histological damage caused by PEG-HoF_3_ NPs through long-term toxicity assessment. Thirty days after injection, the two groups’ mice were sacrificed. The main organs and tissues (heart, liver, spleen, lung, kidney, and muscle) underwent H&E staining for histopathological assessment.

**Figure 4:**
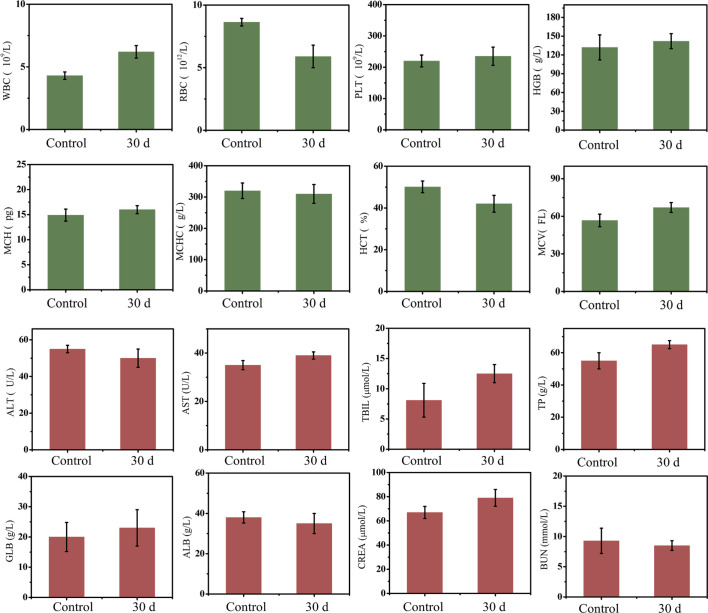
Mice blood testing and biochemical examination, there is no evitable changes in two groups.

Paraffin sections conformed that there was no significant damage or severe inflammation in organs or tissues in either the control group or PEG-HoF_3_ NPs injection group (**Figure 3C**). Encouraged by these results, quantitative analysis of PEG-HoF_3_ NPs potential toxicity was carried out *via* blood testing and biochemical examination. Routine blood tests can be used to observe changes in the distribution of blood cells to judge the condition of mice, and biochemical examination can be used to detect various products of metabolism in blood to show the condition of organs such as liver or kidney. Thirty days after injection, there were no significant abnormalities in the morphology or indices of blood cells **Figure 4**. Metabolism of the main organs also remained stable, which was consistent with the physiological status of the mice. Taken together, the results confirmed that Ho-based nanomaterials offer great potential for bio-application researches due to their high biocompatibility.

### CT Imaging

**Figure 5:**
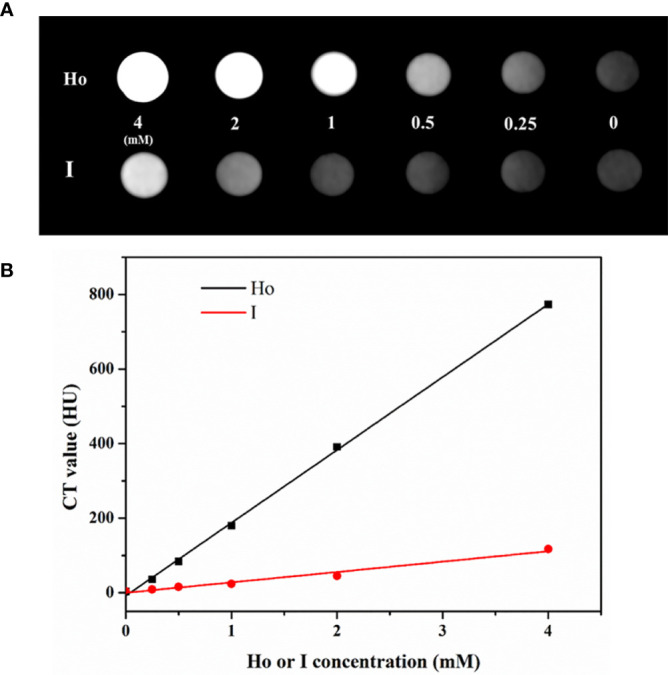
**(A)***In vitro* CT images of PEG-HoF_3_ NPs and Iohexol aqueous solution with different concentration; **(B)** CT value of corresponding tube of A.

As a rare earth element, Ho has an atomic number of 67, which is much higher than iodine’s atomic number of 53. The inherent characteristics of Ho, specifically its high x-ray attenuation, result in the significantly improved imaging ability of Ho-based CAs in CT. The *in vitro* CT imaging results are shown in **Figure 5A**. Different concentrations of PEG-HoF_3_ NPs and Iohexol aqueous solution (0-4 mM) were prepared in Eppendorf tubes and the solutions became brighter as the concentration increased. As shown in **Figure 5B**, the Hounsfield Units (HU) value of PEG-HoF_3_ NPs was significantly higher than that of iodine-based CAs, as we expected, and the HU values for both increased linearly. Therefore, the necessary dose of PEG-HoF_3_ NPs is much lower than that of iodine-based CAs to achieve the same contrast effect *in vivo*, which may reduce the risks of large doses of CAs (39).

**Figure 6:**
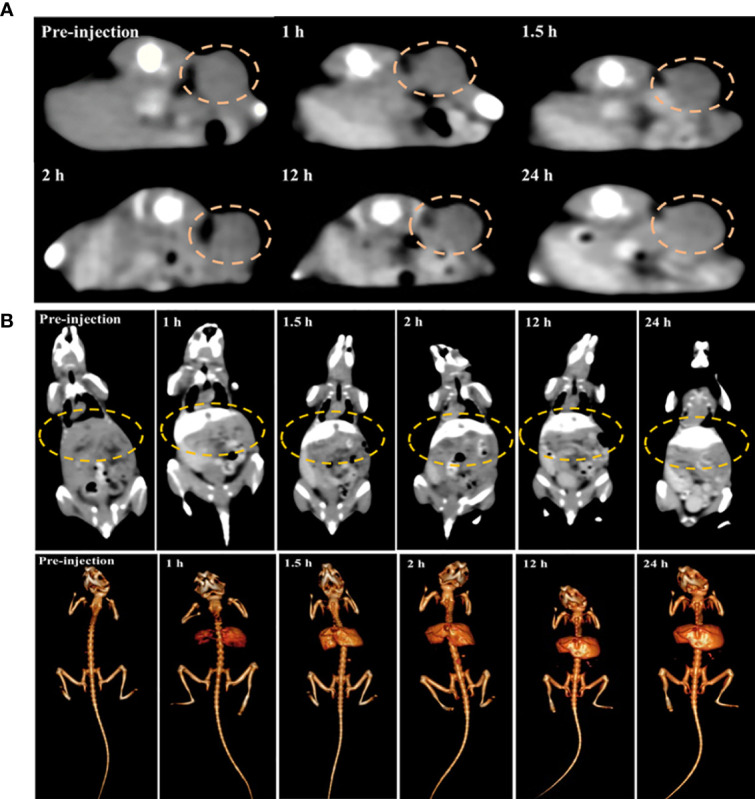
**(A, B)** Changes of tumor site and mouse liver in different time point before versus post-injection of PEG-HoF_3_ NPs.

After cytotoxicity assessment of PEG-HoF_3_ NPs, tumor-bearing Kunming mice were selected to test the CT contrast effect *in vivo*. The distribution of PEG-HoF_3_ NPs was monitored by a CT scanner at different time points. We then compared the images at these time points with the images pre-injection: the brightness of the tumor site was obviously higher after 24 h (**Figure 6A**).

We also tracked the brightness of the liver and kidney after administering PEG-HoF_3_ NPs *via* the tail vein (**Figures 6B** and **S5**). Usually, small molecule iodine CAs are not long-lasting in the liver, which decreases the diagnostic ability of liver disease to some extent. However, as shown in **Figure S5**, the liver of the mice maintained a high contrast effect after 24 h, which likely indicates the uptake of PEG-HoF_3_ NPs by hepatocytes. However, there was no obvious enhancement in the kidney at the time points we used. This may indicate that the uptake of PEG-HoF_3_ NPs by renal cells is limited and that the size and shape of PEG-HoF_3_ NPs prevented its filtration through the kidney. It is possible that the above mechanism can decrease the side effects of CAs on the kidney, and warrants further study.

### T_2_-Weighted MRI

**Figure 7:**
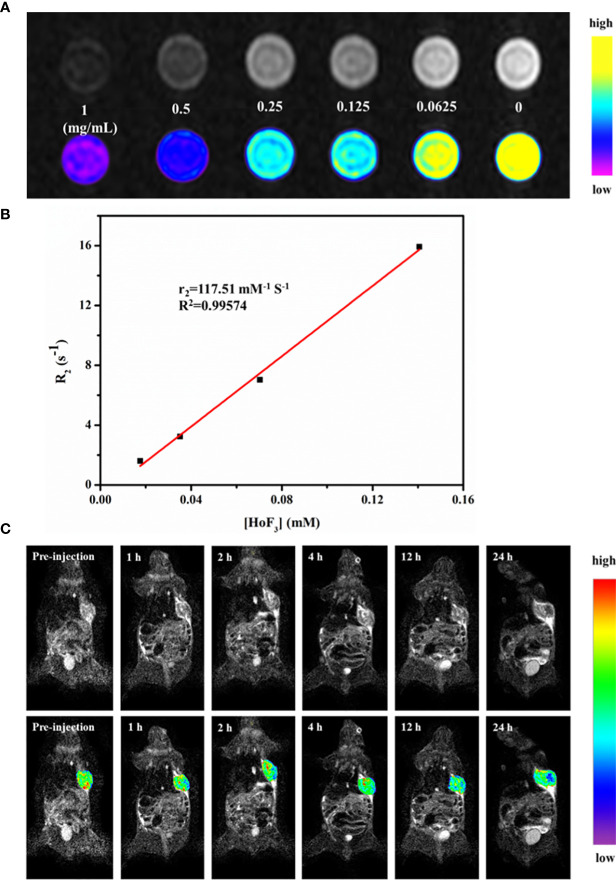
**(A)***In vitro* T_2_-weighted MRI images of PEG-HoF_3_ NPs with different concentration; **(B)** The r_2_ relaxivity plot of PEG-HoF_3_ NPs; **(C)** Signal changes of tumor site in different time point before and post-injection of PEG-HoF_3_ NPs.

To date, there have been few studies on Ho-based MRI imaging. It has been confirmed that Ho-based nanomaterial has T_2_-weighted MRI contrast ability owing to its intrinsic paramagnetism. However, the magnetic resonance relaxivity of NPs can differ due to their size, form, and even synthesis methods. For this reason, we firstly validated the feasibility of PEG-HoF_3_ NPs as a T_2_-weighted MRI CAs *in vitro*. Different concentrations of PEG-HoF_3_ NPs aqueous solution were tested on a clinical MR scanner. T_2_-weighted images of PEG-HoF_3_ NPs aqueous solution (0-1 mg/mL) gradually darkened with an increasing Ho concentration (**Figure 7A**). According to the concentration of PEG-HoF_3_ NPs, the r_2_ relaxivity value of this new contrast agent is 117.51 mM^-1^ S^-1^ (**Figure 7B**). Next, we further explored the potential of PEG-HoF_3_ NPs as negative MRI CAs in tumor-bearing mice. T_2_-weighted MRI was performed on the same scanner at different times (1, 2, 4, 12, 24 h) after intravenous injection of PEG-HoF_3_ NPs (**Figure 7C**). The results showed no significant changes in the signal of the tumor site within 0 to 2 h. However, after 12 h, the signal of the tumor site decreased significantly and a large dark area gradually presented. At 24 h, the negative contrast agent filled the center of tumor site. These results also confirm long term circulation of PEG-HoF_3_ NPs, which will be helpful for improving the detection of tumors at specific places.

## Conclusions

We firstly synthesized a new Ho-based NP *via* a one-pot solvothermal method. The new PEG-HoF_3_ NPs had a uniform size and showed good dispersibility in aqueous solution. Cytotoxicity assessment and histological analysis indicated that the new Ho-based NPs had good biocompatibility and low toxicity for applications *in vivo*. We then further verified the imaging ability of the PEG-HoF_3_ NPs *in vitro* and *vivo*. The results showed that PEG-HoF_3_ NPs had an excellent contrast effect in both CT and T_2_-weighted MRI. Based on this desirable performance, we expect that PEG-HoF_3_ NPs hold great promise for dual-modal imaging and that Ho-based nanomaterials warrant further research.

## Data Availability Statement

The original contributions presented in the study are included in the article/**Supplementary Material**. Further inquiries can be directed to the corresponding authors.

## Ethics Statement

The animal study was reviewed and approved by Laboratory Animal Center of Jilin University.

## Author Contributions

TZ contributed to conception and design of the study. MD and LZ provided the database of mice. ZL contributed to the data of revised manuscript. YL and SS provided the testing instrument and site. TZ wrote the first draft of the manuscript. TG and QY revised and edited the manuscript. All authors contributed to the article and approved the submitted version.

## Funding

This work was supported by the Program of Science and Technology Development Plan of Jilin Province of China (Nos. 20190201218JC), the Health Special Project of Jilin Province Department of Finance (Nos. 2019SCE7025, 2020SCZT088).

## Conflict of Interest

The authors declare that the research was conducted in the absence of any commercial or financial relationships that could be construed as a potential conflict of interest.

## Publisher’s Note

All claims expressed in this article are solely those of the authors and do not necessarily represent those of their affiliated organizations, or those of the publisher, the editors and the reviewers. Any product that may be evaluated in this article, or claim that may be made by its manufacturer, is not guaranteed or endorsed by the publisher.
