# Supplementary material for: Facile Synthesis of Holmium-Based Nanoparticles as a CT and MRI Dual-Modal Imaging for Cancer Diagnosis
Source: Front Oncol. 2021 Aug 26;11:741383. doi: 10.3389/fonc.2021.741383 (PMC8427799; doi:10.3389/fonc.2021.741383)
Supplement: Supplementary file 1 [file DataSheet_1.docx]

**Facile synthesis of Holmium-based nanoparticles as a CT and MRI Dual-modal imaging for Cancer Diagnosis**

**Tianqi Zhang^1,4^, Mo Deng^2^, Lei Zhang^3^, Yang Liu^4,5^, Shuyan Song^4^, Tingting Gong^1*^ and Qinghai Yuan^1*^**

^1^Department of Radiology, the Second Hospital of Jilin University, Changchun, China

^2^Department of Clinical Laboratory, the Second Hospital of Jilin University, Changchun, China

^3^Department of Neurology, the Second Hospital of Jilin University, Changchun, China

^4^State Key Laboratory of Rare Earth Resource Utilization, Changchun Institute of Applied Chemistry, Chinese Academy of Sciences, Changchun, China

^5^University of Science and Technology of China, Hefei, China

*** Correspondence:**Tingting Gong; Qinghai Yuan

gongtt01@jlu.edu.cn; yqh@jlu.edu.cn

**1. Characterization**

Transmission electron microscope (TEM) images were performed on a FEI Tecnai G2 F20 microscope. Field emission scanning electron microscope (FESEM) images were analyzed on a Hitachi S-4800 microscope. X-ray powder diffraction (XRD) of the samples was examined on a D8 Focus diffractometer (Bruker., Germany) using Cu-Ka radiation (0.15405 nm). The concentration of the nanomaterial was obtained by inductively coupled plasma-mass spectrometry (ICP-MS). X-ray photoelectron (XPS) measurements were performed on an ESCALAB-MKII spectrometer (VG Co., United Kingdom). IR measurements were performed on a fourier transform infrared spectrometer (Bruker., Germany). Zeta potential and DLS were analyzed on a Zetasizer Nano (Malvern., England). CT was performed by a 256-slice CT scanner (Brilliance iCT, Philips Healthcare). MR Imaging was performed on a 3.0 T human clinical MRI scanner (Ingenia 3.0T CX, Philips Healthcare).


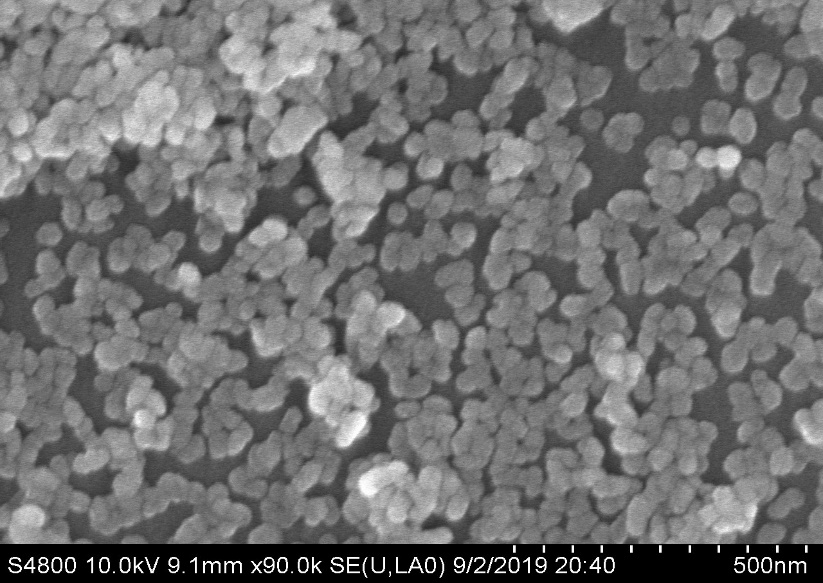


Figure S1. SEM image of PEG-HoF_3_ NPs and the average size is about 38 nm.


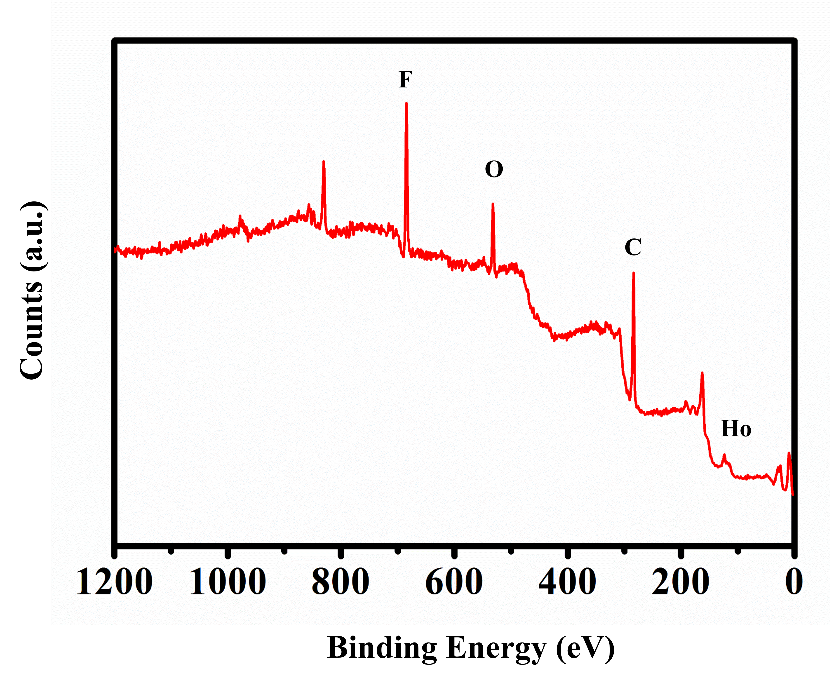


Figure S2. XPS wide scan of PEG-HoF_3_ NPs.





**-OH**

Figure S3. The peak in 3391 cm^-1^ verify the existence of PEG.


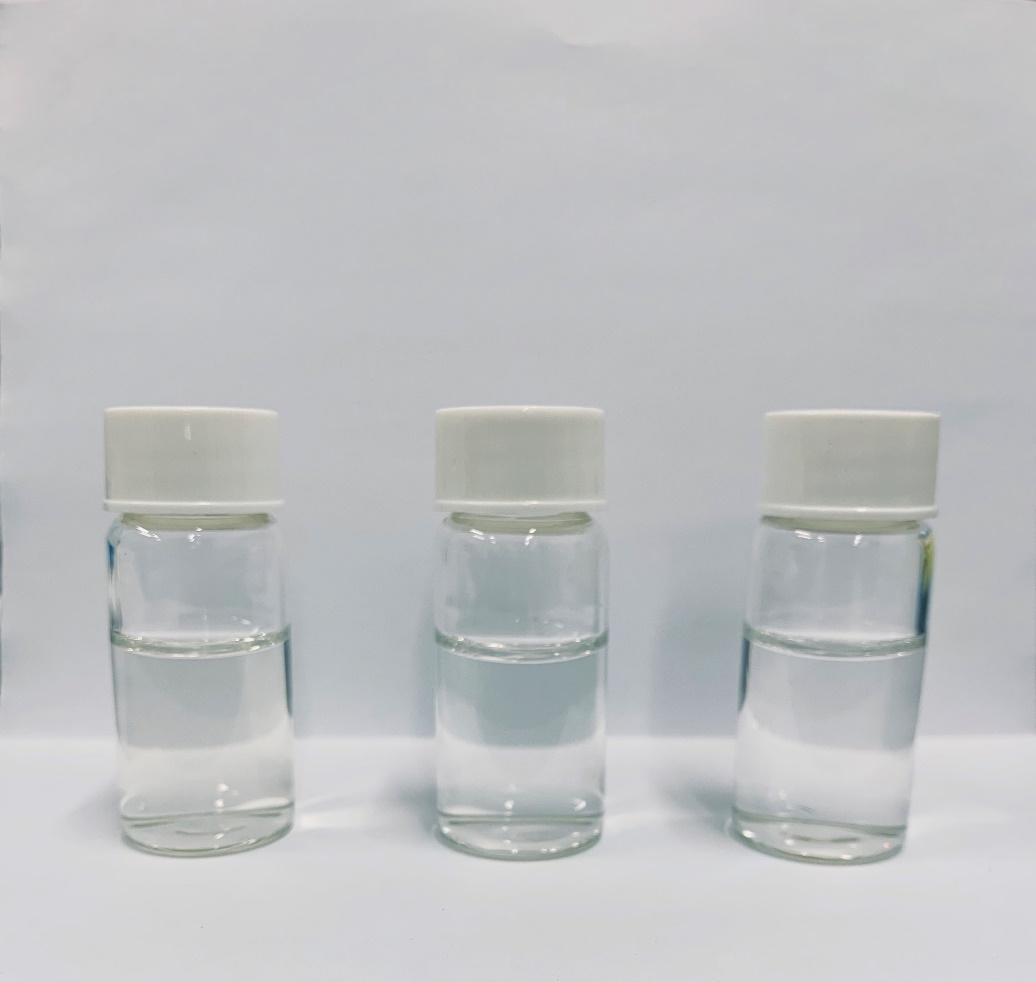


Figure S4. From the left to right are PEG-HoF_3_ NPs in water, normal saline and PBS solution.


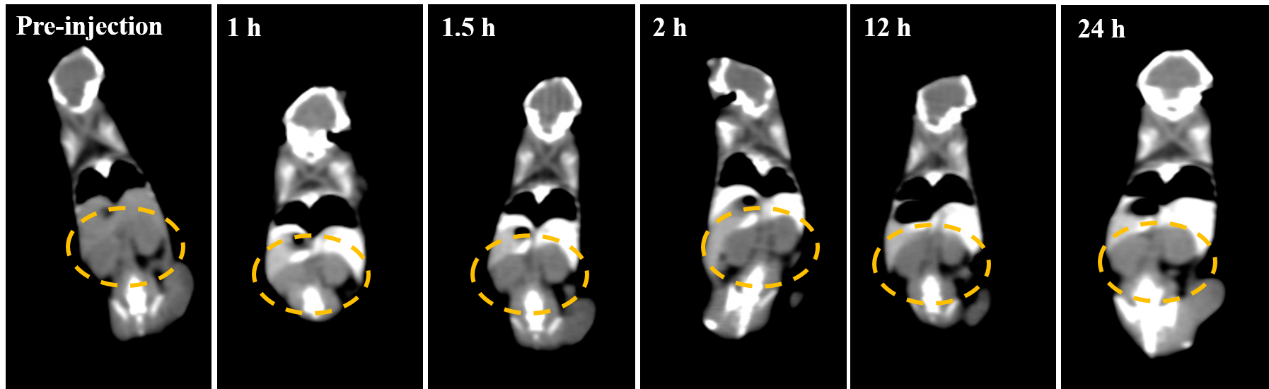


Figure S5. CT images of mouse kidney in different time point after intravenous injection of PEG-HoF_3_ NPs. Yellow circle indicates the kidney.
